# Development of the Wheelchair outcomes Assessment Tool for Children (WATCh): A patient-centred outcome measure for young wheelchair users

**DOI:** 10.1371/journal.pone.0209380

**Published:** 2018-12-26

**Authors:** Lorna Tuersley, Nathan Bray, Rhiannon Tudor Edwards

**Affiliations:** Centre for Health Economics and Medicines Evaluation, Bangor University, Bangor, Gwynedd, United Kingdom; University of Rome, ITALY

## Abstract

**Objective:**

To develop a patient-centred outcome measure (PCOM) for use with children and young people accessing National Health Service (NHS) wheelchair and posture services. Identifying and addressing outcomes of most importance to young wheelchair users (≤ 18 years) will help services maximise the benefits achievable within available resources.

**Methods:**

A mixed-methods approach was used, involving questionnaire surveys and qualitative interviews, and building on previous work identifying how young wheelchair users define health-related quality of life (HRQoL). Framework analysis was used to analyse the interview transcripts. Survey questionnaires seeking views on the importance of a range of outcomes were completed by 21 young wheelchair users or their parents. Subsequent face-to-face interviews with 11 parents or dyads of parents and young wheelchair users explored these responses and identified novel outcomes. Interviewees also scored and recorded satisfaction levels for their key outcomes.

**Results:**

All outcomes proposed in the survey were rated as ‘extremely important’ by at least one respondent, as were additional outcomes uncovered in the qualitative data. In consultation with the service providers and service users, the Wheelchair outcomes Assessment Tool for Children (WATCh) was developed to allow service users and providers to identify, score and monitor individual users’ most important outcomes. The final WATCh tool comprises 16 outcome options, of which service users select five to be monitored. The tool will be used to measure key outcomes identified by service users before and after wheelchair provision.

## Introduction

Providing the right wheelchair at the right time has a profound impact on the holistic wellbeing of children and young people with impaired mobility [1,2], through functional mobility improvement [3], psychosocial development [4] and the advancement of communication skills [3,5,6]. In the UK, the NHS is the largest supplier of wheelchairs and other assistive mobility technology for children; over 60,000 children are registered with NHS wheelchair services in England alone [7]. In 2017, NHS England published a model services specification for wheelchair and posture services [8], incorporating ambitions for wheelchair services identified in the Wheelchair Leadership Alliance’s ‘Right Chair, Right Time, Right Now’ Charter [9]. One of the Core principles includes “a timely, standardised, holistic assessment process with co-produced (with service users/families/providers) outputs and outcome measures…”

Providing mobility equipment which meets individual need in childhood encourages independence [10], limits challenging behaviour [4] and reduces reliance on assistance [3]. Appropriate provision can also reduce pain, risk of complications and improve children’s overall quality of life [11]. Conversely, inappropriate mobility equipment can restrict children’s independence, ability to play and social interaction [12] In order to promote effective and successful paediatric wheelchair interventions in the UK, it is essential that good assessment, training and information are provided by NHS wheelchair services [13] and that appropriate outcomes measures are available to measure benefits.

Although addressing clinical need is an important part of wheelchair provision, young wheelchair users have widely varying needs due to the range of reasons underlying wheelchair use, and comorbidities. Social, developmental and education needs are particularly important when assessing outcomes in wheelchair provision [2], and due to the variation in the needs and abilities of children accessing wheelchair services, it is important that the assessment of wheelchair interventions reflects the needs of individual patients.

Identifying and addressing the outcomes of most importance to young wheelchair users could help wheelchair services to maximise the benefits achievable within available resources. Outcome measures currently in use among rehabilitation specialists are used for determining the overall therapy approach and have a focus on clinical issues (for example the Therapy Outcome Measure [14] or are too complex to routinely deliver in a busy wheelchair service (for example the Canadian Occupational Performance Measure [15]).

Patient Centred Outcome Measures (PCOMs) are designed to focus outcome measurement in healthcare around the needs and priorities of patients—thereby promoting approaches to healthcare which take into account the outcomes which are of most importance to patients [16]. This project was part of a research programme funded by NHS England through Shropshire Clinical Commissioning Group (CCG) to develop PCOMs for use with children and young people. Our aim was to develop the Wheelchair Outcomes Assessment Tool for Children (WATCh); a PCOM designed specifically for NHS paediatric wheelchair and posture services.

## Methods

The objective of the WATCh development project was to identify the main outcomes of importance to young wheelchair users and to then construct a PCOM tool comprising those outcomes. A mixed quantitative and qualitative approach was used, involving questionnaire surveys, semi-structured interviews and piloting of the proposed tool to test usability in practice. The project was approved by the Bangor University Healthcare and Medical Sciences Ethical Committee and the Wales Research Ethics Committee 5, Bangor (REC 17/WA/0078).

Potential participants were identified by the Shropshire NHS Wheelchair and Posture Service based on a broad inclusion criteria: current wheelchair users; aged 18 or under; seen by the service between June 2014 and May 2017. A questionnaire survey and information about the research were sent to 210 potential participants. This was sent directly to those aged 16 years or over, or to their parents/carers if younger. Local patient support groups also advertised the research through social media. The questionnaire survey was developed in partnership between Health Economists from Bangor University (comprising the primary research team) and representatives from the Shropshire CCG, Telford and Wrekin CCG and the Shropshire NHS Wheelchair and Posture Service. Feedback, particularly with regard to readability, was sought from the Telford and Wrekin CCG patient engagement team and a small number of young people.

**Fig 1:**
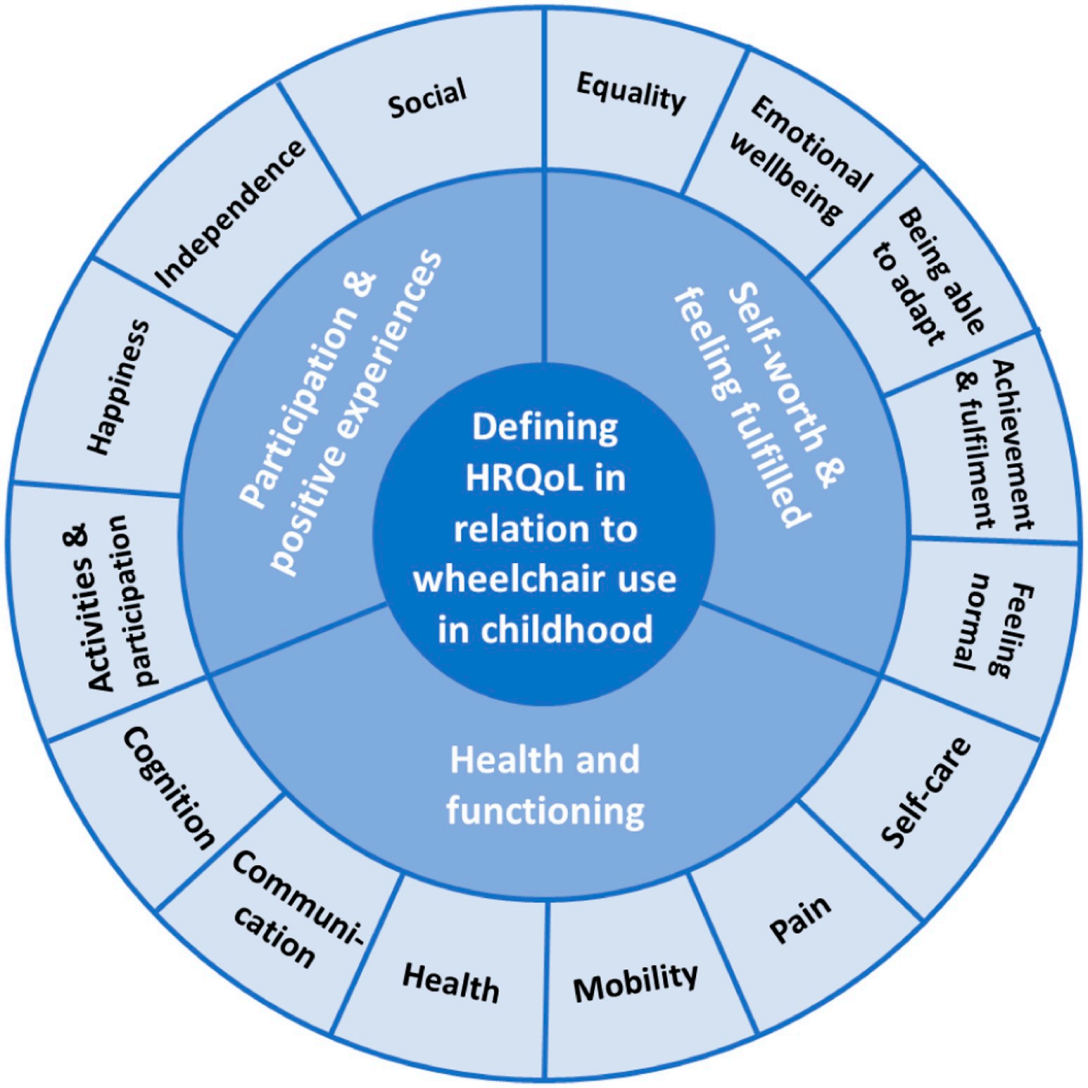
Defining QoL in relation to wheelchair use in childhood: A thematic summary and map [17].

The first part of the questionnaire collected demographic data and information about wheelchair use. In the second part participants were asked to rate the importance of 12 aspects of life (i.e. ‘outcomes’) that a wheelchair could be expected to affect. These pre-defined outcomes were based on previous qualitative research carried out by one of the authors. An exploratory descriptive method using semi-structured qualitative interviews with 11 young wheelchair users and 24 parents of young wheelchair users was used to develop a thematic summary and map of mobility-related Quality of Life (QoL) domains for children and young people (Fig 1). Interviews facilitated participants to consider how they define QOL in relation to mobility impairment, and to reflect on the ability of standard HRQoL measures to represent this definition. Methods and results are reported in full elsewhere [17].

The list of pre-defined outcomes used in the questionnaire survey was refined through discussions between the research team and the wheelchair service and presented in Appendix 1. Participants were permitted to identify up to five ‘other’ outcomes of importance to them, if they felt that these were missing from the pre-defined outcomes. The rating task used a scale from 1 (‘not at all important’) to 5 (‘extremely important’). After completing the rating task, participants were asked to identify their ‘top 3’ outcomes, and for each of these to give a short description of i) what they had hoped their wheelchair would help them achieve; and ii) their actual experience of outcome achievement.

All participants sent a questionnaire survey were invited to take part in a semi-structured interview. Interviews were planned as dyads of young wheelchair users and their parent/carer, and carried out in interviewees’ homes. The aim of the interviews was to further explore participants’ questionnaire responses and their views on desired outcomes. Respondents in eight interviews were also asked to score their top three outcomes in a similar way to that envisaged for final PCOM tool, rating out of 10: how they felt before they got their latest wheelchair (‘Retrospective’); how they felt shortly before they got their latest wheelchair (‘Anticipated’); and finally how they actually felt after using their latest wheelchair for a substantial amount of time (i.e. more than 3 months) (‘Current’). All interviews were carried out by LT, a researcher with experience of qualitative interviewing, and were tape recorded with additional notes taken at the time. Tapes were transcribed verbatim by a professional transcriber. None of the participants were known to the interviewer prior to the interviews, which were arranged by telephone or email.

A ‘framework analysis’ approach [18] was applied to the qualitative data analysis of the interview transcripts, assisted by the software package NVivo. Framework analysis comprises five key stages: familiarisation, identifying a thematic coding framework, indexing, charting and mapping/interpretation. A thematic coding framework was developed in the familiarisation stage, building on the themes identified during the development of the questionnaire. Interview transcripts were then coded line by line during the indexing stage, and inductively coded new themes were incorporated into the framework, until no new themes were identified. During the charting stage themes were then grouped in categories of related codes and finally refined into higher level outcome areas through mapping and interpretation of the findings. Analysis was performed by the interviewer. Although there was no formal second coding, transcripts were also read by NB.

Questionnaire data on the relative importance of outcomes in users’ lists of top 3 outcomes were analysed in order to assess the relevance of each outcome for inclusion in the final PCOM tool. Service staff and local patient engagement groups also provided input. Finally, the quantitative and qualitative data were synthesised to develop a prototype paper-based PCOM tool, aiming to identify the desired wheelchair intervention outcomes for young wheelchair users (or their parent/carer by proxy), and to assess their satisfaction with outcome achievement following receipt of their wheelchair. The resulting prototype PCOM was piloted with a small number of young wheelchair users and/or their parents, and by service staff using the prototype tool with patients at clinic assessment visits. This piloting was primarily undertaken to test usability of the PCOM tool.

**Fig 2:**
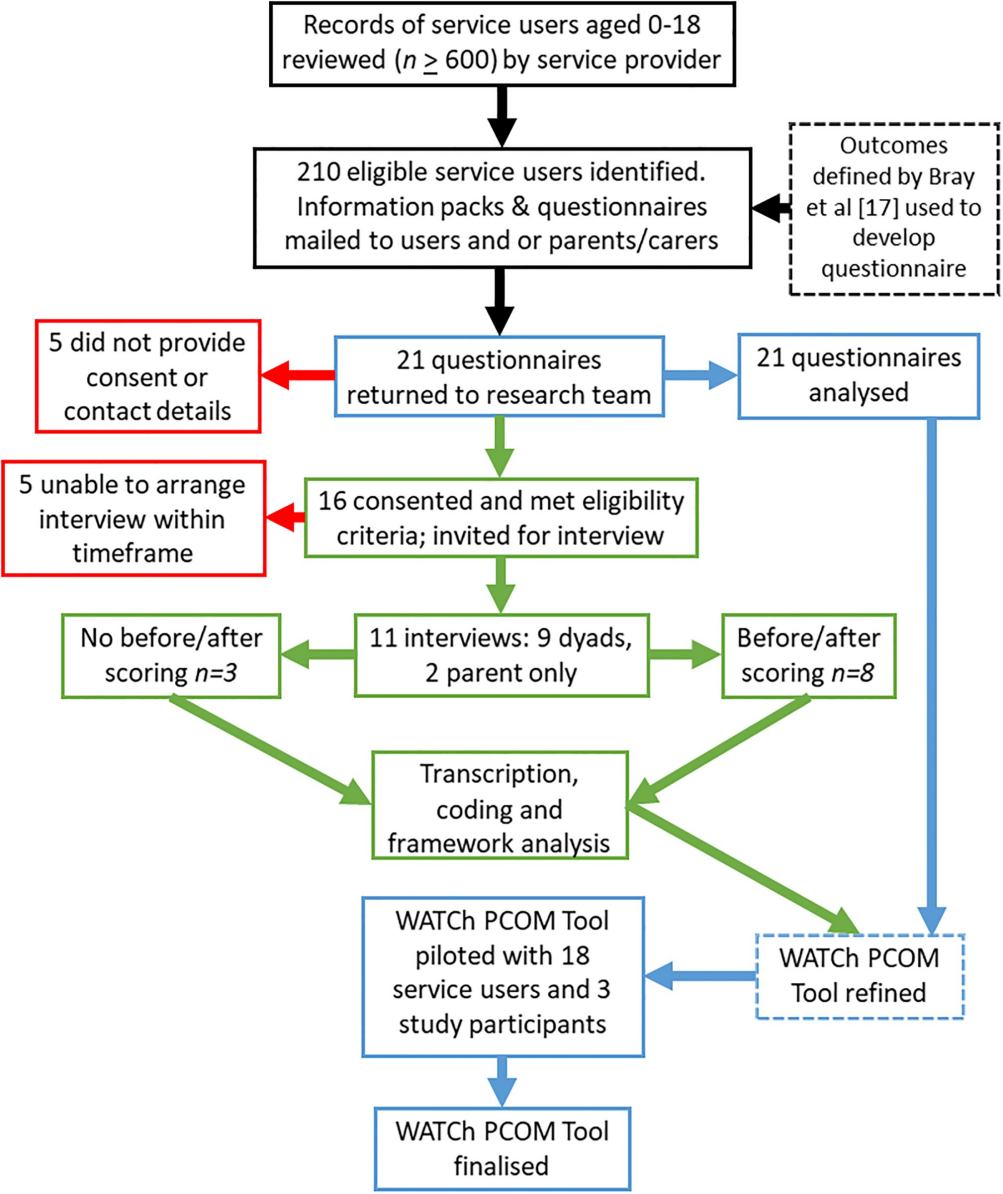
Flow diagram of participants.

A flow diagram of the recruitment and participants at each stage is presented in Fig 2.

## Results

### Survey findings

Summary demographic statistics are shown in Table 1. Twenty-one questionnaires were returned, a response rate of 10%. Although the response rate was not sufficient to allow fully purposive sampling for the interviews, there was a relatively even split of male and female patients, as well as a range of ages and variation in wheelchair use. A number of different underlying conditions necessitating wheelchair use were reported by the patient or their parent/carer.

**Table 1:** Respondent characteristics for children and young people responding to questionnaires and participating in interviews.

		Questionnaires (n = 21)		Interviews (n = 11)	
		Mean	Min—Max	Mean	Min—Max
Age (years)		10.14	3–17	10.82	3–17
		n	%	n	%
**Sex**	**Male**	11	52.4	5	45.5
	**Female**	10	47.6	6	54.5
**No. of wheelchairs in use**	**One wheelchair**	17	81.0	8	72.7
	**Two wheelchairs**	3	14.3	2	18.2
	**One pushchair**	1	4.8	1	9.1
**Primary wheelchair**	**Manual**	18	85.7	8	72.7
	**Powered**	2	9.5	2	18.2
	**Pushchair**	1	4.8	1	9.1
**Frequency of wheelchair use**	**A little of the time**	3	14.3	1	9.1
	**Some of the time**	6	28.6	3	27.3
	**Most of the time**	7	33.3	5	45.5
	**All of the time**	5	23.8	2	18.2
**Condition**	**ADHD/ADS/Other behavioural**	4	19.0	2	18.2
	Heart Condition*	3	14.3	3	27.3
	Cerebral Palsy*	2	9.5	1	9.1
	**Down Syndrome**	2	9.5	0	0.0
	**Other congenital developmental**	6	23.8	4	36.4
	**Neurological**	2	9.5	2	18.2
	**Hypermobility**	1	4.8	0	0.0
	**Chronic regional pain**	1	4.8	0	0.0
	**U/s fatigue, visual difficulties**	1	4.8	0	0.0
	**TOTAL**	22*		12*	

*One patient reported more than one diagnosis

**Fig 3:**
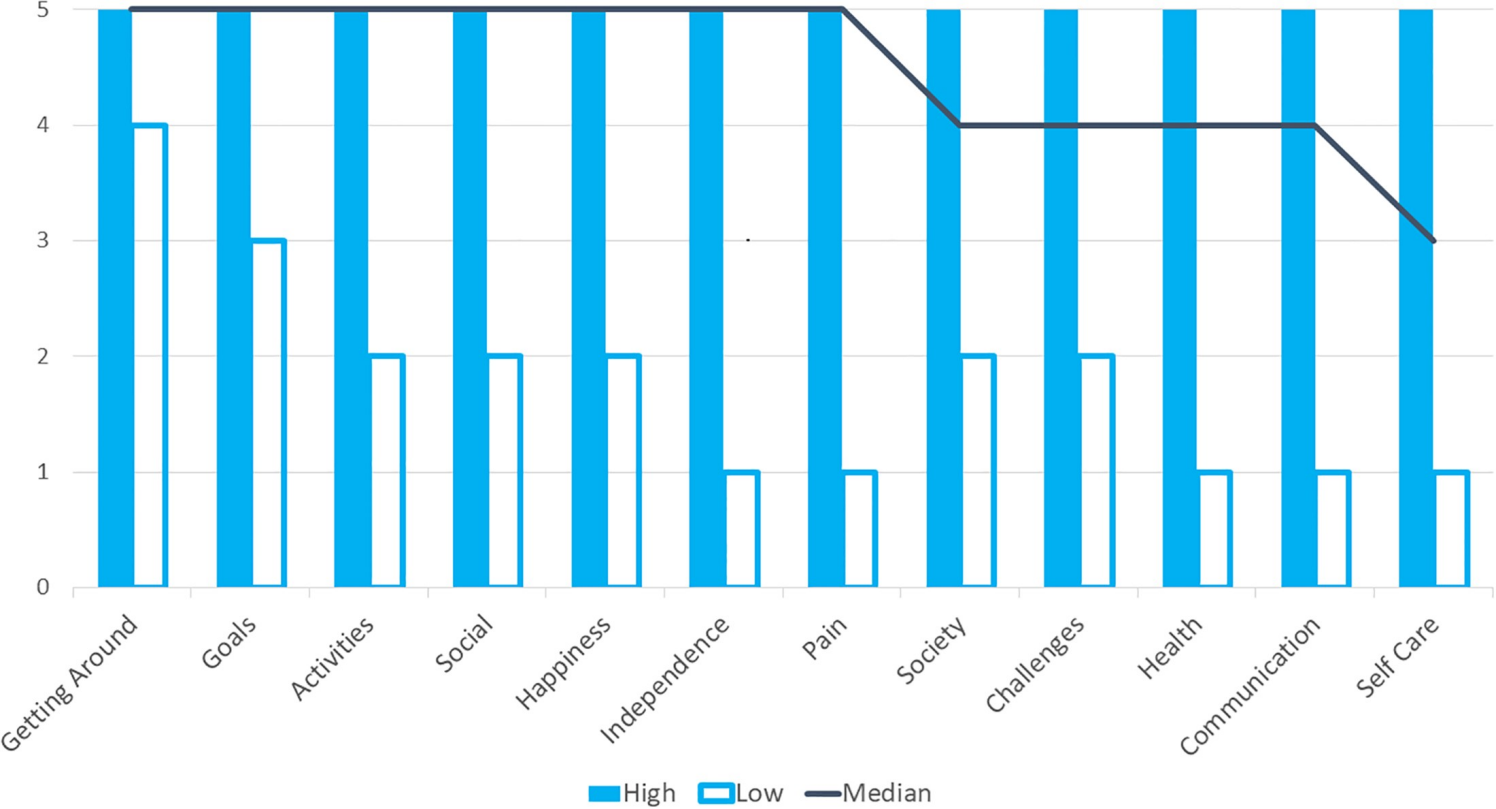
Ranking of importance of outcomes from questionnaire survey (*n* = 21). Rating from 1 Not important to 5 Extremely Important.

The relative ranking of the pre-defined outcomes is presented in Fig 3, based on the median and range of scores. All outcomes were considered appropriate for inclusion in the eventual tool as all were ranked as ‘extremely important’ by at least one respondent.

Twelve respondents described ‘other’ outcomes, most of which related to an aspect already listed, such as ‘getting around’ and ‘activities’. Five novel outcomes were identified in the qualitative data:

- ‘Safety’: including issues around the wheelchair itself, or where the wheelchair prevented users with behavioural issues getting into danger.
- ‘Parent or Carer Wellbeing’: including health issues such as back problems from lifting their child and/or pushing and lifting the wheelchair.
- ‘Energy and Fatigue’: including issues such as tiredness and exhaustion, separate from wider health or pain outcomes.
- ‘Education’: including the ability to access education and the accessibility of school buildings.
- ‘Self-esteem and Confidence’: Including both the positive and negative impacts of wheelchair use on self-image and esteem.

### Interview findings

Sixteen respondents (76%) consented to be interviewed. One was unable to be re-contacted, and arranging interviews within the timescales needed for the project was not possible for four. Eleven interviews took place between July and September 2017, by the end of which point new themes had been exhausted, indicative of saturation being reached. Interviews lasted 45–60 minutes, and involved users from a range of ages, gender and wheelchair usage. The majority of interviews included both a parent (usually the mother) and young wheelchair user. Five young people, all aged at least 11 years, participated fully in their interviews. In six interviews, the views expressed were largely those of the parent/carer due to the absence of the young wheelchair user; reasons for absence included illness or school attendance (*n* = 2); or user unable to communicate due to their condition/age (*n* = 4).

As well as the themes specified in the questionnaire, probing users’ experiences in more depth highlighted the specific reasons for choice of the top outcomes, and uncovered outcomes of importance which had not been considered explicitly. A total of 16 individual outcomes were defined from the survey responses and qualitative data; see Table 2 for the full list of outcomes and illustrative qualitative quotes.

**Table 2:** List of WATCh tool outcome choices and illustrative qualitative quotes.

WATCh outcomes	Example quotes
**Achievement and goals**	*“Well*, *I feel it’s important to overcome*. *Because without her wheelchair*, *she wouldn’t be able to go out and do anything*, *so everything would be a challenge and a difficulty*.*”* ***~*** *“I’m really into photography…I used to go on loads of walks and take loads of photo*. *Now we can go on walks in the wheelchair and it means I can still go on walks and take photos*.*”* ***~*** *“…And you’re going to be doing your Duke of Edinburgh so your wheelchair is going to come in very useful for that*, *for things like the expedition*.*”*
**Activities and fun**	*“With my wheelchair I am give [sic] an outside life in school holidays to enjoy the sunshine when we have some*. *As I can’t walk far as my legs hurt or I collapse*.*”* **~** *“I took him to Disneyland Paris…[he] had a wheelchair seat and he was able to stay in the buggy on the Eurostar*. *Without the buggy*, *he’d have never managed to go there at all*. *It would never have been possible*.*”* ***~*** *“The chair just enables her to not be stopped from doing things*.*”*
**Communication**	*“P*: *It’s helped you do more face-to-face things*, *hasn’t it*? *C*: *Yeah*, *it has*. *P*: *Before*, *she could only communicate online*. *But you could communicate*, *with the wheelchair*, *face-to-face*.*”* **~** *“We’d only been here two or three days and he went outside in his electric wheelchair*, *he went down to the bottom*, *there*, *and started talking to the neighbour next-door*, *went over right to the fence … So it’s great because he can just go and do those things*.*”*
**Education**	*“I definitely wanted to go to college in a wheelchair…And I wanted as much independence as I possibly could get out from it*.*”* **~** *“I find it hard to walk long distances*. *So I tend to use it at school*…*Because it’s a two-site school and after lessons*, *I have to walk a lot through the day*, *and it gets more painful*. *So I tend to use it for that*.*”*
**Energy and fatigue**	*“She knows that she can just go in the wheelchair and it’s not going to cause this horrible fatigue*. *You could just see the weariness on her face*, *that*: *‘It’s too much for me and I can’t do it*.*’ We don’t get half of that now*. *It’s just*, *it’s so much better*.*”* **~** *“He self-propels himself and he’s got quite significant heart defects*, *so he gets tired really quickly*. *He can do it for three*, *four strides*, *then that’s too much*.*”*
**Feeling included**	*“The whole school do the Race for Life at the end of July so [he] does his in his wheelchair…so it means that he is no different to the rest of his peer group*.*”* ***~*** *“Parent [P}*: *[She] has had issues with people saying she’s faking it*, *because they don’t* *understand the condition that one minute she could be ok*, *the next minute she can be really quite poorly with it*. *C*: *And students thinking that our family as a whole or anyone with our condition is faking it*.*”*
**Happiness**	*“She was very pleased*. *She came out beaming*, *smiling…It was more grown up for her*…*She was smiling all the way out of the building really*.*”* ***~*** *Researcher [R]*: *Thinking back to before getting a wheelchair*, *how would you rate feeling happy*? *Child/young person [C]*: *Probably about a four*. *R*: *And now*? *C*: *About ten*.*”*
**Independence**	*“[Before having a wheelchair] I didn’t really have any independence because [parent] just moved me everywhere*, *really*.*”* ***~*** *“I’ve got Lupus and I can’t push her very far unless it’s on flat*. *The idea was*, *she had independence*, *she could maybe go to college*. *But she hasn’t got any independence at all*.*”*
**Managing your condition**	*“We’ve done loads of different trips*…*There’s no way I would be able to take him anywhere without a pushchair*. *Plus*, *when he’s in a PEG feed*, *he needs to be strapped in so that I can do a PEG feed*.*”* ***~*** *“The discomfort comes with the breathing*, *doesn’t it*? *Not being able to breathe and the tiredness*. *She’s got problems with her back*, *now*, *which we need to go to the doctors about today*. *So*, *having the wheelchair helps with that*.*”*
**Moving around**	*“He can’t get around without [wheelchair]*.*”* ***~*** *“[I] couldn’t get around the house properly*. *Now I could go and sit by the back door*. *So it helps in the house and out*.*”* ***~*** *“She wouldn’t do half of what she does without that wheelchair*. *She would basically be housebound a lot of the time*.*”*
**Pain and discomfort**	*“Without it*, *at some point*, *I wouldn’t have been able to go out with the family*, *really*, *because I’d just be in constant pain with my ribs and my hips*. *And my knees*. *It’s all the knock-on effect*, *isn’t it*?*”* ***~*** *“She had a plastic back brace fitted which pushed her forward*. *So to me*, *she wasn’t sitting as comfortably in it as she could have been*, *until it was adjusted*. *That’s my biggest bugbear is when you need an appointment*, *you need it pretty quickly and you shouldn’t have to be waiting*.*”*
**Parent or carer wellbeing**	*“Not hurt mummy/daddy’s backs to carry me”* **~** *“P*: *If I’m having a bad day*, *we have to stay in the house because I can’t take the weight of your wheelchair*. *C*: *Yeah*, *because it’s a lot*. *It’s heavy*.*”* **~** *“The handles are in the wrong place…Pushing a pushchair*, *the handles are usually flat or angled*, *whereas a wheelchair*…*It’s an unnatural position*. *To be pushing her up hills*, *because she’s quite a weight now*, *it’s very uncomfortable*.*”*
**Safety**	*“[What] he has done is tipped it over backwards*. *That’s been a bit of a problem*.*”* ***~*** *“And on one particular occasion*, *[her] gentleman who used to bring her home*, *he didn’t quite do it properly…And it went back and [she] hit her head on the concrete and we had a little trip to A&E*. *Luckily*, *it was just a cut*.*”* **~** *“I use it mainly in busy areas*, *where there’s risk with traffic*. *Because he hasn’t got any sense of danger so he would run into the road and things like that*.*”*
**Self-care**	*“‘Helping you to look after yourself*, *for example*, *get washed…That’s not relevant because I clean him*.*”* **~** *“If he’s in his chair*, *he can get to the toilet…Which makes things easier*.*”*
**Self-esteem and confidence**	*“He’d had a few comments from his friends and as soon as that happened*, *[he] wasn’t coming to school and that had a knock-on effect to his health*. *So*, *how the wheelchair looks is a big deal*.*”* **~** *“When I’m out with my friends…There would be a point where I would get too tired*…*And they’d have to push me and I just don’t really want that*. *I’d just rather be able to just go out with them…Would be a different story if I was able to push myself the whole time*.*”*
**Social-life**	*“I was very*, *very pleased because with the manual [wheelchair]… I didn’t have much of a good relationship with my friends*.*”* ***~*** *“There is an indirect benefit to her social life*, *in that if she uses the wheelchair to do certain activities*, *she’s not too tired*, *then*, *to be able to meet up with her friends*. *Whilst she doesn’t use it directly with her friends*, *she doesn’t waste her energy doing other things*.*”*

The eight interviewees who were asked to allocate satisfaction scores ‘before’ (‘Retrospective’), prior to receipt of the chair (‘Anticipated) and after receiving (‘Current’) their wheelchair indicated that they all understood the process. One user lacked detailed recall when scoring the ‘before’ status, but this is unlikely to be an issue in practice when using real-time scoring. Table 3 shows scoring by individual user. All showed some improvement on their top outcomes after receipt of their chair compared to before getting one, the vast majority achieving at least 50% of their maximum score. This confirmed that a range of levels of satisfaction could be determined using this approach, even among a small number of respondents. The range of positive to negative scores obtained comparing the ‘Current’ and ‘Anticipated’ scores also highlighted that several users had unmet expectations.

**Table 3:** Interviewees’ retrospective scoring of top outcomes before and after wheelchair provision (*n* = 8).

Respondent ID	Retrospective^1^ Max = 30	Anticipated^2^ Max = 30	Current^3^ Max = 30	% Max Current^4^	Current minus Anticipated	Current minus Retrospective
**012**	15	30	30	100	0	15
**015**	18.5	30	30	100	0	11.5
**016**	8	15.5	29	97	13.5	21
**008**	6	30	24	80	-6	18
**007**	3	24	19.5	65	-4.5	16.5
**014**	NA*	30	15.5	52	-14.5	NA
**005**	0	10**	5	50	-5	5
**018**	0	30	5	17	-25	5

^1^ Outcome score before receiving most recent wheelchair

^2^ Outcome score respondent hoped to achieve before receiving most recent wheelchair

^3^ Actual outcome score at present (i.e. after provision of most recent wheelchair)

^4^ Current total outcome score compared to maximum possible

*Respondent did not score Retrospective outcomes

**Respondent only selected one outcome, maximum total score is therefore 10

### WATCh PCOM design and piloting

A two-page paper-based prototype ‘WATCh Assessment tool’ was designed using the findings from the survey/interviews and with input from the wheelchair service team, the Telford and Wrekin CCG Patient Engagement Lead for readability and members of the West Midlands wheelchair managers group.

In Part A of the WATCh tool, users select their top 5 outcomes from 16 pre-specified outcomes (listed in Table 2), based on the original survey plus the additional outcomes identified in the qualitative data. In Part B, users rank their top 5 outcomes in order of importance. They are then asked to describe what they want to achieve, and indicate their present level of satisfaction for each outcome, before receiving their new wheelchair. The scoring was simplified to 5 levels from ‘very dissatisfied’ to ‘very satisfied’, in line with other measures such as the EQ-5D-5L [19] and the Child Health Questionnaire [20]. ‘Smiley’ faces have been used to indicate levels of satisfaction for younger service users. A separate follow-up tool, Part C, has been developed to follow-up outcomes 3–6 months after provision of a wheelchair.

In order to test usability, Parts A and B were piloted by members of the wheelchair services team in clinic, and in telephone interviews with two of the three parents not previously exposed to the scoring at interview, and a parent who returned a late consent to participate. Table 4 presents the pilot results. All but two pre-specified outcomes (‘self-care’ and ‘communication’) were selected by at least one respondent within their top 5, highlighting the range of individual preferences among service users. The new areas of ‘education’, ‘safety’ and ‘parent or carer wellbeing’ as well as ‘energy and fatigue’ were included in the top 5 by over 19% of respondents. No new outcomes were noted as ‘Other’. All possible satisfaction scores were used by at least one respondent, confirming that the form may be suitable for clients with both well-met and unmet needs.

**Table 4:** Pilot usability data: Outcomes ranked by inclusion in Top 5, satisfaction scored from 1 (very dissatisfied) to 5 (very satisfied).

Outcome list	No. of times chosen in Top 5	% of respondents (*n = 21)*	% of all Top 5 choices (*n = 101)*	Median satisfaction*	Mean satisfaction*
**Activities and fun**	13	62	13	3	2.85
**Moving around**	11	52	11	3	2.80
**Education**	11	52	11	3	3.20
**Social life**	10	48	10	3	3.00
**Pain and discomfort**	10	48	10	3	3.10
**Safety**	9	43	9	3	3.25
**Energy and fatigue**	9	43	9	2	2.22
**Managing your condition**	8	38	8	2.5	2.63
**Independence**	6	29	6	3	2.60
**Happiness**	5	24	5	3	3.00
**Parent or carer wellbeing**	4	19	4	3.5	3.25
**Feeling included**	3	14	3	3	2.67
**Self-esteem and confidence**	1	5	1	4	4.00
**Achievement and goals**	1	5	1	4	4.00
**Self-care**	0	0	0	0	0
**Communication**	0	0	0	0	0
**Other**	0	0	0	0	0

The WATCh assessment tool was confirmed to be straightforward to use by the majority of service staff involved in the pilot, and was left unchanged. Although no participant in the pilot selected ‘self-care’ or ‘communication’ within their top 5, it was felt important to continue to include these outcome choices given the importance noted by some of the respondents during the development phase. Similarly it was considered important to allow users to have an opportunity to state anything of importance under ‘other’ if they felt an outcome was not covered within the predefined outcome categories. Positive feedback included the ability to encourage discussion and record patient requirements and expectations. Concerns included the time taken for completion, although the median time taken was 10 minutes. After minor revision to layout, the tool was finalised and made available in paper form and electronically (please see appendices for the paper version and http://cheme.bangor.ac.uk/watch-tool for the electronic version).

## Discussion

WATCh is the first patient-centred outcome measure developed specifically for young wheelchair users, filling a gap in service commissioning to promote child wellbeing and social development. The simple before and after scoring system allows service providers to ascertain how well individual’s desired outcomes are being achieved, both for individual users and by outcome across service users.

The WATCh tool is designed to be used when assessing a child’s requirements for a new wheelchair, and then repeated three to six months afterwards, to assess any change in outcomes. The tool can be completed via a number of means, including online, paper and via telephone, to suit each service and service user. The tool can be completed in a clinic or home setting, and can be completed independently by the patient.

At the time of commissioning this project, while there were several outcomes tools available to and in use by therapists and assistive technology providers within the NHS, none were specifically aimed for use with children and young adults requiring a wheelchair to obtain and assess achievement of prospectively identified patient-centred outcomes.

Those aimed at users of wheelchairs or other assistive devices have largely evaluated pre-determined aspects. The Quebec User Evaluation of Satisfaction with Assistive Technology (QUEST) [21] evaluates levels of satisfaction with aspects of the service or the technology. While it is relevant to wheelchair users, it only captures satisfaction with what has already been provided, and is aimed at adults.

The Functioning Everyday with a Wheelchair (FEW) [22] (also previously known as the Functional Evaluation in a Wheelchair instrument [23]), is a self-report scoring system on ability to carry out specific tasks, aimed at an adult population. The Wheelchair Users Functional Assessment (WUFA) [24] also evaluates ability to undertake a number pre-determined activities in an observed performance-based tool, and is also developed for adults. Neither assess the more social needs of the user. A measure that has been developed from the child’s perspective, and is assessed by self-report, is the Activities Score for Kids (ASK) measure [25]. However the items are pre-determined and focus on functional ability. The Psychosocial Impact of Assistive Devices (PIADS) [26, 27], focuses on functional independence, well-being and quality of life of the patient but also uses predetermined measures and was developed for adults.

Other tools in general use by therapists include the Therapy Outcome Measures (TOM) [14], a general tool for rehabilitation professionals to assess patients at entry and exit points from an episode of care and at intermediate points as appropriate. Patients are assessed and scored against predefined areas using pre-coded levels of achievement. Adaptations of the TOM with specific levels of achievement have been developed for different clinical conditions or situations, including for certain children-specific situations. None are specific to wheelchair use although an ‘Environmental Aids’ specific tool is in development with adaptations to the ‘Activity performance’ levels.

A number of tools aim to determine outcomes defined by or in collaboration with the users, but have not been aimed at children or wheelchair users. The Canadian Occupational Performance Measure (COPM) [15] is intended for use by occupational therapists with patients using semi-structured interviews to identify, rank in importance and rate performance and satisfaction with aspects of their life. It has been reported to have been adapted for and used with children [28]. Similarly, the Goal Attainment Setting (GAS) measure [29] can be used with clients with different problems and therapy approaches, identifying high priority goal areas, and agreeing specific and measurable indicators of progress. Levels of achievement are then scored relative to an expected outcome level. This has also been used in paediatric research [28]. Both are patient-centred but take time to administer, and thus are unlikely to be suitable for time-constrained clinical practice.

Acknowledgement that there was “…no existing tool which can provide individualized goal-orientated measure of outcome after wheelchair provision”, led to the development of the Wheelchair Outcome Measure (WhOM) [30] in Canada. Clients nominate key areas of participation inside and outside the home; rate their importance, and are asked to rate their level of satisfaction with each, at assessment and reassessment. It also includes an assessment of sitting posture and comfort and space for feedback on other issues and information on completion of the form. It has recently been adapted for use with young people as the WhOM-YP [31]. While the overall aim is similar to that of the WATCh tool, it is more lengthy and complex to deliver. Some clients may find it difficult to state which participation outcomes are important to them without some kind of assistance. The WATCh tool indicates outcomes likely to be of most importance which may help the user, but allows for specifics to be documented, and also allows space for anything else important to be selected if not covered by the list.

Problems with the postal service were noted by some respondents, which may have contributed to the low response rate, and which meant that the number of users able to provide input to the development of the tool within the timeframe was lower than hoped for. However, the participants included a range of ages, underlying conditions and type/level of wheelchair use. The outcomes initially proposed were based on previous work on wheelchair users’ needs with 11 young wheelchair users and 24 parents of wheelchair users by Bray et al [17], and a further 19 users and their families were exposed to the WATCh tool at the pilot stage.

Review of the WATCh tool is ongoing. We are currently seeking feedback from a wide range of service providers on the utility of the tool and its implementation, with a view to make revisions if required. Further assessment of the WATCh tool’s reliability and validity is required, but could not be carried out within the limited budget and timescales of this initial PCOM development study. Additional funding is being sought for continued analysis and validation. Supplementary work will be needed to translate the tool into other languages, and to adapt the tool for use with adult wheelchair users. The WATCh tool could, in theory, be used in a wide range of contexts, including in cost-effectiveness analyses as a natural unit of effect to compare costs and outcomes. PCOMs take an almost opposite approach to many forms of traditional outcome measurement, which are typically designed to be unchanging and universal, while PCOMs are very much about tailoring outcome measurement to the individual. The need to focus on individual patients is particularly important in wheelchair provision, as individual needs guide every aspect of the intervention. Comparability across individuals is challenging, as individual outcome profiles will almost certainly be different for each patient, thus the overall scores will reflect different measures of effect. This raises an important question about how we measure outcomes, and whether assessment should focus on outcomes which are relevant to the majority or to the individual. A combination of clinical measures and PCOMs could ensure that interventions meet the needs of the patient, whilst providing the necessary information for clinicians and therapists to tailor interventions to the individual.

## Conclusion

In conclusion, the project achieved the aim of developing a novel, patient-centred outcome measure, the WATCh tool, suitable for use with children and young people accessing NHS wheelchair services. Young wheelchair users across a wide range of ages and clinical needs can select outcomes of most importance to them and express their desired achievements for each outcome. For service providers, the WATCh tool helps patients to focus on achievable outcomes and allows a degree of comparability across patients. The tool should be applicable to wheelchair services across the UK and other countries. In addition to potentially improving the monitoring and assessment of wheelchair interventions for young wheelchair users, the development of the WATCh tool could inform the development of novel PCOMs in other service areas. While formal costing and quality of life measurements were not feasible within this study, the findings should support future work addressing cost-effectiveness of wheelchair provision. The WATCh tool is free to use and available from http://cheme.bangor.ac.uk/watch.

### Ethical approval and consent to participate

The project was approved by the Bangor University Healthcare and Medical Sciences Ethical Committee and the Wales Research Ethics Committee 5, Bangor (REC 17/WA/0078). The study conformed to the tenets of the Declaration of Helsinki. Written informed consent was obtained from all participants prior to participation in the study. Consent forms were completed by all young adults over 16, and all parents participating in interviews. If the child was under 16 they completed assent forms and parents completed proxy forms as appropriate.

## Supporting information

S1 Appendix Pre-defined outcomes list from initial survey. (PDF)

S2 Appendix WATCh Assessment Tool. (PDF)

S3 Appendix WATCh follow-up tool. (PDF)

## Abbreviations

ASK: Activities Score for Kids
CCG: Clinical Commissioning Group
CPOM: Canadian Occupational Performance Measure
FEW: Functioning Everyday with a Wheelchair
GAS: Goal Attainment Setting measure
HRQoL: Health Related Quality of Life
NHS: National Health Service
PACS: Parents and users Council (Shropshire)
PCOM: Patient Centred Outcome Measure
PIADS: Psychosocial Impact of Assistive Devices
PODS: Parents Opening Doors (Telford)
QoL: Quality of life
QUEST: Quebec User Evaluation of Satisfaction with Assistive Technology
REC: Research Ethics Committee
TOM: Therapy Outcome Measure
U/s: unspecified
WATCh: Wheelchair outcomes Assessment Tool for Children
WhOM: Wheelchair Outcome Measure
WhOM-YP: Wheelchair Outcome Measure—Young Person
WUFA: Wheelchair Users Functional Assessment
