# Supplementary material for: Development of the Wheelchair outcomes Assessment Tool for Children (WATCh): A patient-centred outcome measure for young wheelchair users
Source: PLoS One. 2018 Dec 26;13(12):e0209380. doi: 10.1371/journal.pone.0209380 (PMC6306207; doi:10.1371/journal.pone.0209380)
Supplement: S1 Appendix — (PDF) [file pone.0209380.s001.pdf]

APPENDIX 1

Initial outcomes as used in the Questionnaire

Please tick the box to say how important the following things about your wheelchair are to you:

1. Helping you to take part in activities and play?

Not important ☐ *Somewhat important* ☐ *Important* ☐ *Very important* ☐ *Extremely important* ☐

2. Letting you do more without help from other people?

Not important ☐ *Somewhat important* ☐ *Important* ☐ *Very important* ☐ *Extremely important* ☐

3. Helping you have a better social life?

Not important ☐ *Somewhat important* ☐ *Important* ☐ *Very important* ☐ *Extremely important* ☐

4. Helping you to get around?

Not important ☐ *Somewhat important* ☐ *Important* ☐ *Very important* ☐ *Extremely important* ☐

5. Helping you to achieve your goals and the things that are important to you?

Not important ☐ *Somewhat important* ☐ *Important* ☐ *Very important* ☐ *Extremely important* ☐

6. Helping to reduce any pain and discomfort you may have?

Not important ☐ *Somewhat important* ☐ *Important* ☐ *Very important* ☐ *Extremely important* ☐

Continued on next page

Continued

7. Helping you to look after yourself (for example to get washed and dressed by yourself)?

Not important ☐ *Somewhat important* ☐ *Important* ☐ *Very important* ☐ *Extremely important* ☐

8. Helping you feel part of wider society? (People other than family and friends)

Not important ☐ *Somewhat important* ☐ *Important* ☐ *Very important* ☐ *Extremely important* ☐

9. Helping to improve your overall health?

Not important ☐ *Somewhat important* ☐ *Important* ☐ *Very important* ☐ *Extremely important* ☐

10. Helping you to communicate with and interact in other ways with people?

Not important ☐ *Somewhat important* ☐ *Important* ☐ *Very important* ☐ *Extremely important* ☐

11. Helping you to overcome challenges and difficulties in life?

Not important ☐ *Somewhat important* ☐ *Important* ☐ *Very important* ☐ *Extremely important* ☐

12. Helping you to feel happy?

Not important ☐ *Somewhat important* ☐ *Important* ☐ *Very important* ☐ *Extremely important* ☐

Are there any other reasons why your/your child's wheelchair is important? If yes, please write them below:

13.____________________________________

14.____________________________________

15.____________________________________

16.____________________________________

17.____________________________________
