# Supplementary material for: Development of the Wheelchair outcomes Assessment Tool for Children (WATCh): A patient-centred outcome measure for young wheelchair users
Source: PLoS One. 2018 Dec 26;13(12):e0209380. doi: 10.1371/journal.pone.0209380 (PMC6306207; doi:10.1371/journal.pone.0209380)
Supplement: S2 Appendix — (PDF) [file pone.0209380.s002.pdf]

Appendix 2

Shropshire Community Health NHS
NHS Trust

WATCh Assessment Form

(Wheelchair outcomes Assessment Tool for Children)

Information for wheelchair users and parents/carers

We are using this form as part of your assessment, to help us to find out what goals you have in relation to your new wheelchair. The form has two parts:

- Part A lists some areas of your life which your wheelchair might be able to help you with. Please decide which are the **FIVE** most important areas to you
- Part B then asks you to score how satisfied or happy you are now with each of the top **FIVE** areas you chose in Part A

Once you've had your new wheelchair for a few months, we will ask you to score your top five list again to see if there has been any improvements. If you have any questions about the form, or problems filling it in, please let the person doing your assessment know. See below for an example of how to complete this form.

Example of how to complete Part A

Area of your life	How your wheelchair could help	Top 5
1. Activities and fun	Help you to take part in activities and fun	✓
2. Independence	Help you to do more without help from other people	
3. Social life	Help you to spend time with your friends and family	
4. Moving around	Help you to get around inside and outside of the house	✓
5. Pain and discomfort	Help to reduce your pain or discomfort related to posture	
6. Self-care	Help you to wash and dress yourself	
7. Feeling included	Help you to feel part of wider society	
8. Managing your condition [illegible]	Help to manage your condition and avoid health problems [illegible]	✓

Tick your top FIVE areas

Example of how to complete Part B

Transfer answers from Part A

Top 5 (in order)	Area	What you want to achieve or feel	How satisfied or happy you are with this area of your life
1 (most important)	*Energy and fatigue (no.15)*	*Feel less tired when using my wheelchair*	Very Dissatisfied / **X Dissatisfied** / Neutral / Satisfied / Very Satisfied
2	*Moving around (no.4)*	*Be able to move around school by myself*	Very Dissatisfied / Dissatisfied / Neutral / **X Satisfied** / Very Satisfied
3	*Happiness (no.11)*	*Feel less worried and upset*	Very Dissatisfied / Dissatisfied / **X Neutral** / Satisfied / Very Satisfied
4	*Activities and fun (no.1)*	*Be able to go to the shops with friends*	**X Very Dissatisfied** / Dissatisfied / Neutral / Satisfied / Very Satisfied
5	*Pain and discomfort (no.8)*	*Improve my posture and reduce pain*	Very Dissatisfied / **X Dissatisfied** / Neutral / Satisfied / Very Satisfied

Patient name:__________________________DOB:_____/____/_____ NHS No.:________________

Assessor: ____________________________________Date:____/____/__________

Completed by: *(please tick)* **Patient** ☐ **Parent/Carer** ☐ **Assessor** ☐

PART A

Below are a list of different areas of your life which your new wheelchair could make a difference to. Please look at this list and place a tick in the box next to the top 5 most important areas for you. Please make sure you only chose FIVE areas. If there's something missing from the list, you can write it in the space at the bottom.

Area of your life	How your wheelchair could help	Top 5
1. Activities and fun	Help you to take part in activities and fun	
2. Independence	Help you to do more without help from other people	
3. Social life	Help you to spend time with your friends and family	
4. Moving around	Help you to get around inside and outside of the house	
5. Pain and discomfort	Help to reduce your pain or discomfort related to posture	
6. Self-care	Help you to wash and dress yourself	
7. Feeling included	Help you to feel part of wider society	
8. Managing your condition	Help to manage your condition and avoid health problems	
9. Communication	Help you to communicate and interact with others	
10. Education	Help you to go to school and learn	
11. Happiness	Help you to feel happy and free from worry	
12. Safety	Help you to feel safe and secure	
13. Parent or carer wellbeing	Help your parent or carer to stay happy and healthy	
14. Self-esteem and confidence	Help you to feel more self-confident	
15. Energy and fatigue	Help you to feel more energetic and less tired	
16. Achievement and goals	Help you to achieve the things that are important to you	
17. Anything else?<br>Please tell us here:		

Patient name:____________________ DOB:___/___/___ NHS No.: __________

Assessor: ____________________ Date:___/___/___

PART B

In the boxes below, please write your top 5 areas from Part A in the order of their importance. You can also add a bit more information about what you want to achieve. So, if you chose 'activities and fun' in your top 5, you could say what you want to achieve, like starting a new sport or hobby. Please then rate how satisfied or happy you are now with your experience of the 5 areas, on a scale from 'very dissatisfied' to 'very satisfied'. For instance, if you aren't happy with how much pain you have at the moment, you might tick 'dissatisfied'.

Top 5 (in order)	Area	What you want to achieve or feel	How satisfied or happy you are with this area of your life
1 (most important)			○ Very Dissatisfied ○ Dissatisfied ○ Neutral ○ Satisfied ○ Very Satisfied
2			○ Very Dissatisfied ○ Dissatisfied ○ Neutral ○ Satisfied ○ Very Satisfied
3			○ Very Dissatisfied ○ Dissatisfied ○ Neutral ○ Satisfied ○ Very Satisfied
4			○ Very Dissatisfied ○ Dissatisfied ○ Neutral ○ Satisfied ○ Very Satisfied
5			○ Very Dissatisfied ○ Dissatisfied ○ Neutral ○ Satisfied ○ Very Satisfied
