# Supplementary material for: Development of the Wheelchair outcomes Assessment Tool for Children (WATCh): A patient-centred outcome measure for young wheelchair users
Source: PLoS One. 2018 Dec 26;13(12):e0209380. doi: 10.1371/journal.pone.0209380 (PMC6306207; doi:10.1371/journal.pone.0209380)
Supplement: S3 Appendix — (PDF) [file pone.0209380.s003.pdf]

Appendix 3

Shropshire Community Health NHS

NHS Trust

WATCh Follow-up Form

(Wheelchair outcomes Assessment Tool for Children)

Information for wheelchair users and parents/carers

You may remember that we gave you a form to complete before you got your new wheelchair, to help us find out what goals you had in relation to your new wheelchair.

In Part B of the form we asked you to tell us a bit more about the 'Top 5' most important areas of your life and to score how satisfied or happy you were with them before getting your new wheelchair.

Now you have had your wheelchair for a little while, we would like you to score these 'Top 5' again to see if your new wheelchair has helped.

If you have any questions about the questionnaire, or need help filling it in, please speak to your therapist or clinical team.

Patient name:____________________________________DOB:____/____/______ NHS No.:__________________

Assessor: ____Date:____/____/__________

PART C Follow up

Below are the top 5 areas you chose when you filled in the questionnaire at your assessment. Now that you have had your wheelchair for a little while, please rate how satisfied or happy you are now with these 5 areas, on a scale from 'very dissatisfied' to 'very satisfied'. For instance, if you aren't happy with how much pain you have at the moment, you might tick 'dissatisfied'.

Top 5 (in order)	Area	What you wanted to achieve or feel
1 (most important)		
2		
3		
4		
5		

How satisfied or happy you are with this area of your life

Very Dissatisfied ○ — Dissatisfied ○ — Neutral ○ — Satisfied ○ — Very Satisfied ○

Very Dissatisfied ○ — Dissatisfied ○ — Neutral ○ — Satisfied ○ — Very Satisfied ○

Very Dissatisfied ○ — Dissatisfied ○ — Neutral ○ — Satisfied ○ — Very Satisfied ○

Very Dissatisfied ○ — Dissatisfied ○ — Neutral ○ — Satisfied ○ — Very Satisfied ○

Very Dissatisfied ○ — Dissatisfied ○ — Neutral ○ — Satisfied ○ — Very Satisfied ○
